# Comprehensive Analysis of Novel Synergistic Antioxidant Formulations: Insights into Pharmacotechnical, Physical, Chemical, and Antioxidant Properties

**DOI:** 10.3390/ph17060690

**Published:** 2024-05-27

**Authors:** Sorinel Marius Neacșu, Magdalena Mititelu, Emma Adriana Ozon, Adina Magdalena Musuc, Izabela Dana Maria Iuga, Bogdan Nicolae Manolescu, Simona Petrescu, Jeanina Pandele Cusu, Adriana Rusu, Vasile-Adrian Surdu, Eliza Oprea, Dumitru Lupuliasa, Ioana Andreea Popescu

**Affiliations:** 1 Department of Pharmaceutical Technology and Biopharmacy, Faculty of Pharmacy, “Carol Davila” University of Medicine and Pharmacy, 6 Traian Vuia Street, 020945 Bucharest, Romania; sorinel-marius.neacsu@drd.umfcd.ro (S.M.N.); dumitru.lupuliasa@umfcd.ro (D.L.); andreea-ioana.popescu@umfcd.ro (I.A.P.); 2 Department of Clinical Laboratory and Food Safety, Faculty of Pharmacy, “Carol Davila” University of Medicine and Pharmacy, 6 Traian Vuia Street, 020945 Bucharest, Romania; magdalena.mititelu@umfcd.ro (M.M.); izabela-dana-maria.iuga@mst.umfcd.ro (I.D.M.I.); 3 Institute of Physical Chemistry—Ilie Murgulescu, Romanian Academy, 202 Spl. Independentei, 060021 Bucharest, Romania; simon_pet@icf.ro (S.P.); jeanina@icf.ro (J.P.C.); arusu@icf.ro (A.R.); 4 “C. Nenitescu” Department of Organic Chemistry, Faculty of Applied Chemistry and Science of Materials, National University for Science and Technology Politehnica Bucharest, 1–7 Gh. Polizu Street, 011061 Bucharest, Romania; bogdan.manolescu@upb.ro; 5 Department of Science and Engineering of Oxide Materials and Nanomaterials, Faculty of Chemical Engineering and Biotechnologies, National University for Science and Technology Politehnica Bucharest, 1–7 Gh. Polizu Street, 011061 Bucharest, Romania; adrian.surdu@upb.ro; 6 Department of Microbiology, Faculty of Biology, University of Bucharest, 1–3 Portocalilor Way, 060101 Bucharest, Romania; eliza.oprea@g.unibuc.ro

**Keywords:** oxidative stress, antioxidant action, biotin, resveratrol, coenzyme Q10, quercetin

## Abstract

(1) Background: Oxidative stress plays a pivotal role in the pathogenesis of various diseases, including neurodegenerative disorders, cardiovascular diseases, cancer, and diabetes, highlighting the pressing need for effective antioxidant interventions. (2) Methods: In this study, we aimed to develop and characterise two novel antioxidant formulations, F3 and F4, as therapeutic interventions for oxidative stress-related conditions. (3) Results: The physicochemical characterisation, preformulation analysis, formulation, preparation of filling powders for capsules, capsule content evaluation, and antioxidant activity assessment of the two novel antioxidant formulations were assessed. These formulations comprise a combination of well-established antioxidants like quercetin, biotin, coenzyme Q10, and resveratrol. Through comprehensive testing, the formulations’ antioxidant efficacy, stability, and potential synergistic interactions were evaluated. (4) Conclusions: The findings underscore the promising potential of these formulations as therapeutic interventions for oxidative stress-related disorders and highlight the significance of antioxidant interventions in mitigating their progression.

## 1. Introduction

Oxidative stress is caused by a series of environmental factors including pathogens, UV rays, the action of herbicides, and pollution. Dietary factors are also one of the main causes of oxidative stress. Many of the drugs contain toxins that can cause the occurrence of processes that are the basis of the triggering of oxidative stress [1,2,3]. These can, in some cases, inhibit the functionality of DNA or the production of enzymes, both crucial in the stages of glycolysis and oxidation [4,5]. Limiting these activities can cause the production of a large amount of free radicals [6,7]. Free radicals cannot simply be eliminated, they act on cell membranes, modifying DNA structures, which results in the deformation or death of the respective cells (the result is what we see in the mirror, premature aging and a state of poor health) [8,9]. Oxidative stress, resulting from an imbalance between the production of reactive oxygen species (ROS) and the antioxidant defence system, has been implicated as a key contributor to the pathogenesis of these disorders. Notable conditions associated with or influenced by oxidative stress encompass neurodegenerative diseases, cardiovascular diseases, cancer, diabetes, chronic inflammatory diseases, and age-related disorders [10,11,12,13,14,15,16,17,18,19,20,21,22]. On the other hand, oxidative stress arises from an imbalance between free radical generation and the body’s antioxidant defence mechanisms. This imbalance can lead to cellular damage, impacting proteins, DNA, and cellular functions [23,24,25,26,27,28,29]. The interplay between neuroplasticity and oxidative stress is intricate. However, elevated or persistent oxidative stress can disrupt neuroplasticity. It can cause cellular damage, interfere with signaling pathways critical for adaptive changes, and potentially impair the brain’s ability to adapt and learn [30,31].

Genetic mutations denote alterations in the DNA sequence arising from diverse factors, encompassing environmental influences, intrinsic cellular processes, or hereditary predispositions. These mutations can affect gene and protein functionalities, leading to variations in cellular mechanisms and operations. Oxidative stress itself can induce DNA damage and mutations, potentially exacerbating cellular dysfunction and genetic variations [32,33,34]. Certain genetic conditions are linked to compromised antioxidant pathways or impaired DNA repair mechanisms, heightening susceptibility to oxidative damage. Conditions such as familial amyotrophic lateral sclerosis (ALS) or specific hereditary cancers exemplify associations between mutations affecting cellular defences against oxidative stress [35,36]. Furthermore, oxidative stress-induced DNA damage can prompt mutations in pivotal genes governing cellular regulation, contributing to the onset of diverse ailments, including cancer, neurodegenerative disorders, and age-related conditions [37,38,39,40,41,42,43].

A balanced diet is the best treatment against oxidative stress, thanks to the antioxidants found in fresh fruits and vegetables, which have the ability to annihilate the unhealthy surplus of free radicals. The most important sources of antioxidants are found in nature, in complex forms, and never alone. The polyvitamin complexes provided by fruits and vegetables are very beneficial for the whole body [44,45]. Strategies aimed at mitigating oxidative stress through lifestyle modifications, antioxidant-rich dietary interventions, regular physical exercise, stress management, and targeted medical therapies focusing on antioxidant pathways hold promise in managing or attenuating the risk of these disorders. Further research into understanding the intricate role of oxidative stress in the pathogenesis of these conditions is crucial for devising tailored therapeutic approaches aimed at alleviating oxidative damage and preventing disease progression [46,47,48,49]. To reduce oxidative damage, it is very important to avoid sources of oxidants (cigarettes, alcohol, foods with a poor nutritional index, stress, etc.) or toxic substances (contaminated foods, polluted environments) and to increase the consumption of food rich in antioxidants [50,51].

In this study, two antioxidant formulations, F1 and F2, comprising quercetin, biotin, coenzyme Q10, and resveratrol, in different proportions were subjected to extensive physicochemical characterisation and preformulation analysis to elucidate their composition and stability. Capsule content evaluation was conducted to assess the uniformity of dosage and confirm the presence of active ingredients within each capsule. Antioxidant activity, a critical parameter for assessing the therapeutic potential of antioxidant formulations, was evaluated using appropriate assays to measure radical scavenging activity, lipid peroxidation inhibition, and total antioxidant capacity. The novelty of this study lies in the integration of the synergistic combination of quercetin, biotin, coenzyme Q10, and resveratrol. While each of these compounds has been individually studied for its antioxidant properties, the synergistic interactions between them within the formulations represent a novel approach to combating oxidative stress. By harnessing the complementary mechanisms of action of these compounds, the formulations offer the potential for enhanced antioxidant efficacy compared to single-agent interventions. Additionally, the comprehensive characterisation and evaluation of the formulations’ physicochemical properties, formulation processes, capsule content, and antioxidant activity contribute to the novelty of this research. Literature research has primarily focused on their individual effects, with limited exploration of their combined synergistic actions within formulations. By integrating these compounds in different combinations and proportions into two distinct antioxidant formulations, F3 and F4, this study advances the state of the art by exploring novel combinations of antioxidants tailored for the prophylaxis and treatment of oxidative stress-related conditions.

## 2. Results

### 2.1. ATR-FTIR Spectroscopy

**Figure 1:**
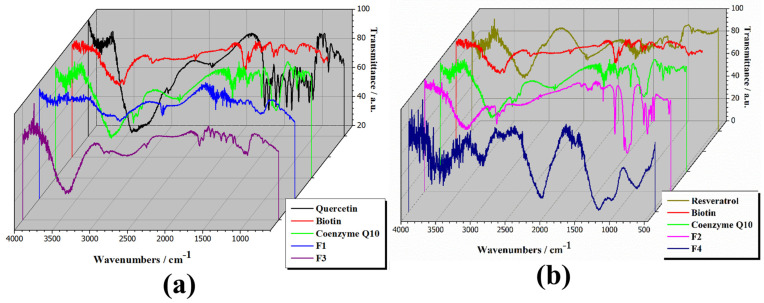
FTIR spectra of analysed compounds (**a**) quercetin, biotin, coenzyme Q10, F1, and F3; (**b**) resveratrol, biotin, coenzyme Q10, F2, and F4, in the range of 4000–450 cm^−1^.

Figure 1 displays the ATR-FTIR spectra of individual components (quercetin, biotin, coenzyme Q10, and resveratrol), formulations with active ingredients (F1 and F2), and final capsule formulations with excipients (F3 and F4).

The ATR-FTIR spectrum of quercetin, depicted in Figure 1a—black line, reveals its characteristic bands [52]. Stretching of O-H groups appeared at 3406 and 3283 cm^−1^, with bending of the phenolic O-H group observed at 1379 cm^−1^. The aryl ketonic C=O stretch absorption was observed at 1666 cm^−1^. Stretching of the C=C aromatic ring manifested at 1610, 1560, and 1510 cm^−1^. In-plane bending of C–H in the aromatic hydrocarbon was detected at 1317 cm^−1^, while out-of-plane bending occurred at 933, 820, 679, and 600 cm^−1^. Additionally, bands at 1263, 1200, and 1165 cm^−1^ were attributed to C–O stretching in the aryl ether ring, C–O stretching in phenol, and C–C=O–C stretching and bending in ketone, respectively. The ATR-FTIR spectrum of biotin (red line from Figure 1) shows the main bands, namely: 3386 cm^−1^—the hydroxyl absorptions; 2931 cm^−1^—CH_2_ group stretching and deformation modes; 1639 cm^−1^—C–O deformation band; 1481 cm^−1^—C=O stretch; 1316 cm^−1^ and 1269 cm^−1^—deformation of skeletal vibrations of C–C moieties [53]. The ATR-FTIR spectrum of coenzyme Q10 (from Figure 1—green line) showed the main absorption bands: at 1646.7 cm^−1^ attributed to C=O; at 1607.0 cm^−1^ attributed to C=C; and at 1446.8 cm^−1^ attributed to C-H bending [54]. The ATR-FTIR spectrum of resveratrol (Figure 1b) showed a broad absorption band at 3200~3500 cm^−1^ attributed to O-H functional groups (phenolic and hydroxyl groups) [55]; other peaks which are revealed between 1440 and 1590 cm^−1^, at 1375 cm^−1^, at 1145 cm^−1^, and 965 cm^−1^ are characteristic of functional groups of resveratrol such as benzene ring, aromatic ring carbon–carbon double-bond vibration, and C-O stretching vibration [56,57]. The peak corresponding to C-C stretching vibration is shown at 1105 cm^−1^, and at 1010 and 965 cm^−1^ are the peaks attributed to bending vibrations of the trans olefinic C=C [58].

The capsule formulations exhibit diminished peak intensities and the disappearance of characteristic peaks of the active ingredients, indicating potential interactions between the compounds. However, the overall spectral profile and characteristic peaks of the final capsule formulation closely resemble those of the formulations without excipients, with only a reduction in peak intensities. This suggests that the excipients primarily function as fillers, superdisintegrants, and lubricants, exerting minimal influence on the structure of the resulting compounds.

### 2.2. X-ray Diffraction Analysis

**Figure 2:**
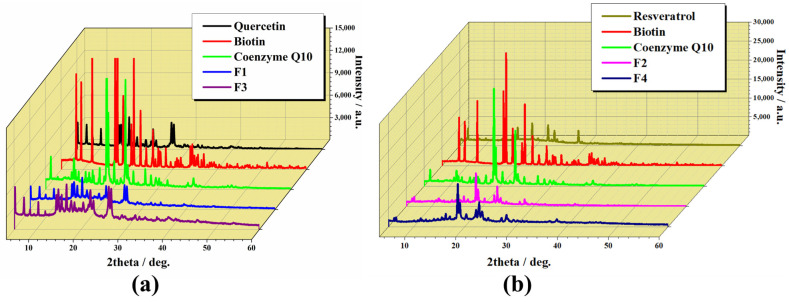
XRD analysis of the individual components and formulations (without and with excipients): (**a**) quercetin, biotin, coenzyme Q10, F1, and F3; (**b**) resveratrol, biotin, coenzyme Q10, F2, and F4, in the range of 2θ = 5–60°.

Figure 2 displays the XRD patterns of all analysed compounds: active ingredients, formulations with active ingredients (F1 and F2), and formulations with excipients (F3 and F4).

The XRD spectra of bioactive compounds (quercetin, biotin, coenzyme Q10, resveratrol) showed numerous defined diffraction peaks within the range of 5° to 40°, proving their crystalline structure [59]. The characteristic peaks of pure quercetin (Figure 2a) predominantly occur at 2θ values ranging from 6.3° to 7.3°, 10.8°, 12.5°, 13.6° to 14.3°, 15.7° to 18.0°, 24.4°, and 27.3°. These peaks signify the crystalline portion of quercetin [60]. Biotin (Figure 2—red line) exhibited nine characteristic crystal peaks at 2θ values of 8.3°, 9.4°, 11.9°, 17.6°, 18.9°, 21.3°, 22.7°, 28.4°, and 34.3°, along with numerous smaller peaks between 30° and 60°. Consequently, the degree of crystallinity of biotin was notably high [61]. Resveratrol exhibited a distinct crystal morphology at a diffraction angle of 2θ (Figure 2b). Within the range of 10° to 30°, strong characteristic diffraction peaks were evident, specifically at 13.41°, 16.54°, 19.36°, 22.54°, 23.75°, 25.39°, and 28.45°, underscoring its elevated crystallinity [62]. The main diffraction peaks of coenzyme Q10 were detected at 2θ = 11.3, 18.5, 20.2, 22.7, and 23.3 (Figure 2) [54]. These findings align with existing literature, indicating a well-defined crystalline structure and organised atomic arrangement for the active ingredients. However, analysis of the capsule formulations revealed a decrease in peak height and number, with the main characteristic peaks disappearing, suggesting a reduction in crystallinity compared to the pure ingredients. This reduction in crystallinity implies interactions between the active ingredients and the excipients used in the capsules, as anticipated. Importantly, these interactions did not adversely affect the active ingredients. Moreover, the XRD results confirmed the reduction in the crystalline state of the ingredients in the final formulation due to the processing method employed during preparation.

### 2.3. Thermal Analysis

**Figure 3:**
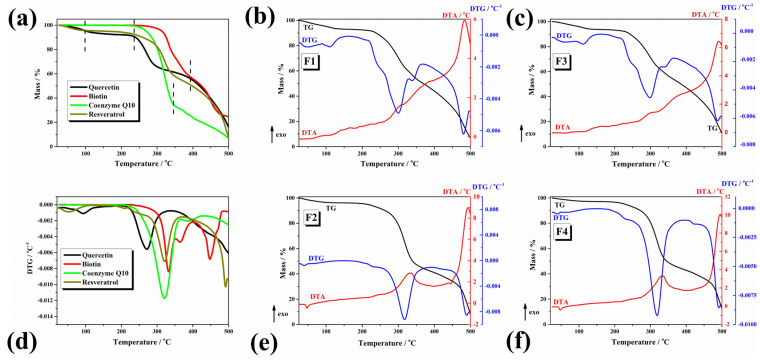
Thermal curves of the individual components and formulations (without and with excipients): (**a**) TG curves of active ingredients quercetin, biotin, coenzyme Q10, resveratrol; (**b**) F1 (TG/DTG—DTA); (**c**) F3 (TG/DTG—DTA); (**d**) DTG curves of active ingredients quercetin, biotin, coenzyme Q10, resveratrol; (**e**) F2 (TG/DTG—DTA) and (**f**) F4 (TG/DTG—DTA); (Thermal conditions: air atmosphere at a flow rate of 80 mL min^−1^ with 10 °C/min heating rate).

Figure 3 shows the thermal curves (TG/DTG) of all analysed compounds: active ingredients, formulations with active ingredients (F1 and F2), and formulations with excipients (F3 and F4).

The TG curves of active ingredients exhibit the following thermal behaviour: biotin and coenzyme Q10 show only two major thermal events between 200 and 500 °C, being thermally stable until 200 °C; quercetin and resveratrol show three thermal events: (i) the mass loss can be ascribed to the loss of water; (ii) the last two thermal events are attributed to the combustion of organic components from the samples which occurs from 200 to 250 °C and 250 to 500 °C for both compounds. The thermal behaviour of the F1 and F2 formulations which incorporate only the active ingredients (quercetin, biotin, and coenzyme Q10 for F1 and biotin, coenzyme Q10, and resveratrol for F2) shows mostly the same trend of their thermal decomposition: (i) an initial mass loss until 140 °C for F1 and until 100 °C for F2 due to the loss of volatile compounds (water, solvents); (ii) the second mass loss is a complex one composed by two decompositions steps between 190 and 380 °C for F1 and 180 and 414 °C for F2, which can be ascribed to the combustion of organics. The thermal decomposition events reported for the formulations with excipients (F3 and F4) resemble the thermal behaviour of the formulations which contain only the active compounds with differences in the onset of thermal decomposition and the final residue. If the residue at 500 °C for F1 is 7.45% and for F2 is 9.8%, the corresponding formulations with excipients show a final mass of 11.7% for F3 and 13% for F4, respectively. Taking into account that the excipients are the same and in the same concentration, the different thermal behaviour is due to the presence of quercetin in F3 and resveratrol in F4 [63,64]. The thermal characteristics of the active ingredients and the F1, F2, F3, and F4 formulations and the decomposition steps, respectively, are presented in Table 1.

### 2.4. SEM Analysis

The pure individual active components, formulations with active ingredients, and formulations with excipient SEM morphologies were observed in order to confirm the morphological changes in final capsule formulations.

**Figure 4:**
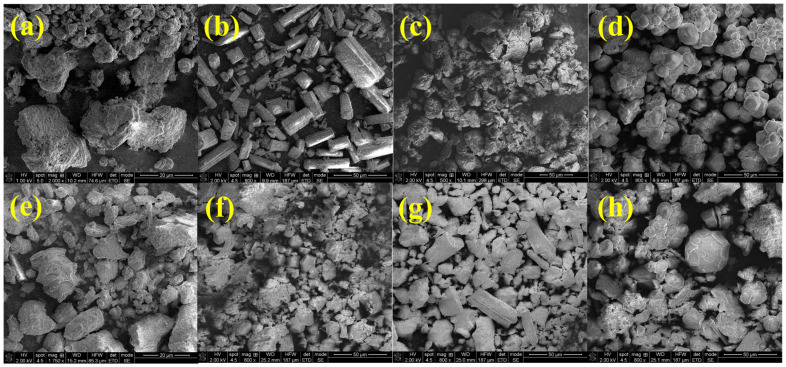
SEM micrographs of (**a**) quercetin; (**b**) biotin; (**c**) coenzyme Q10; (**d**) resveratrol; (**e**) F1; (**f**) F2; (**g**) F3; (**h**) F4, at magnifications in the range of 500× and 2000×.

From Figure 4a, quercetin presents an SEM trip-like structure, characterised by a smooth surface lacking distinct features [65]. The SEM image of biotin shows a needle-like crystal structure (Figure 4b). The SEM morphology of coenzyme Q10 (Figure 4c) was observed as a plate shape and crystalline agglomerates [54]. Resveratrol generally has spherical particles with relatively uniform sizes, and few particles are in an aggregated state (Figure 4d) [62]. In contrast, the SEM images of F1 and F2 formulations (Figure 4e,f) reveal the emergence of a novel structure. The complex appears as extremely fine and irregular crystals, showcasing a noticeable reduction in particle size and a partial loss of crystalline properties compared to the initial components. The SEM analysis confirmed the components’ presence and yielded insights into particle shape and size. The captured SEM micrographs of the F3 and F4 formulation (Figure 4g,h) demonstrate the inability to discern the individual component structures, underscoring a robust interaction between active components and excipients.

### 2.5. Preformulation and Formulation Studies of the Capsules

**Figure 5:**
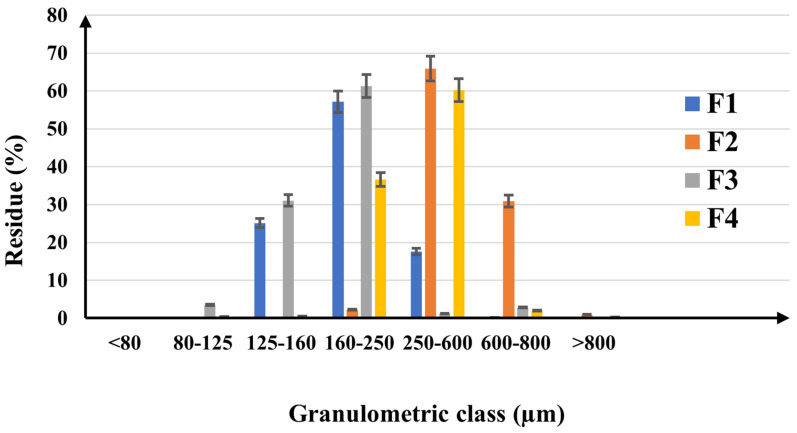
The particle size distribution of the active ingredient’s mixtures (F1 and F2) and the powders for capsule filling (F3 and F4).

Figure 5 shows the histogram representing the particle size distribution for the active ingredient’s mixtures and the powders for capsule filling. All determinations were performed in triplicate. The results are expressed as mean ± SD (standard deviation).

There is a clear difference between the formulations. Firstly, among the active ingredients’ mixtures, F1 has fine particles in the 125–600 µm range with the highest proportion (57.16%) between 160 and 250 µm; at the same time, the vast majority of F2 particles are larger than 250 µm with most in the 250–600 µm interval. The smaller particles in F1, which are found in the 125–160 µm size range, obviously belong to quercetin, while resveratrol is responsible for the coarser particles in F2. Secondly, there is a surprising reduction in the particle size after incorporation of the excipients in both formulations. The main characteristics of small particles in F1 and large particles in F2 remain, but a broader particle size distribution was found for F3 and F4. F3 contains the largest amount of particles (61.29%) in the narrow range of 160–250 µm, and most of the particles in F4 have sizes between 250 and 600 µm. Both active ingredient mixtures have a high humidity content of 5.32% in F1 and 6.88% in F2, which proves that resveratrol contributes an extensive moisture to the powders. After adding the excipients, the moisture content significantly decreases to 2.76% in F1 and 4.04% in F2.

According to Eur. Pharm. [66] criteria, the CI and HR values determined for F1 and F2 are representative of materials with poor flowability, which are not appropriate for the uniform filling of hard capsules. F2, with a CI of 38% and an HR of 1.6, showed lower flow properties than F1 with better values for CI (24%) and HR (1.3), probably due to the presence of resveratrol in the formulation (Table 2). Both bulk density and tapped densities are very different between the active ingredient mixtures, suggesting the significant impact of quercetin and resveratrol on the volumetric characteristics of the powders. Resveratrol is obviously decreasing the flowability and increasing the compactness beyond a reasonable level. By adding the excipients to the composition, only a slight reduction in bulk densities but a drastic decrease in tapped density was noticed. The influence of the excipients is clear, in a positive sense, reducing the values of CI and HR to 19% and 1.2 in F3 and 23% and 1.3 in F4, respectively, which proves that there were certain physical interactions among the particles. F3 and F4 display significantly improved mechanical properties compared to the initial materials, upgrading the flowability and compactness attributes from very weak to moderate, which is suitable for the consistent filling of the hard gelatin capsules. The difference between the two formulations remains, and F3 has better volumetric properties than F4, so it can be expected to behave better during the capsule-filling process.

F1 and F2 powders flowed uniformly only when the 15 mm nozzle was used at a stirring speed of 15 rpm, confirming the poor flowability of active ingredients’ mixtures. As already expected, F2 with a 1.838 g/s flow rate exhibited poor properties compared to F1 with a flow rate of 2.118 g/s. Conversely, a noticeable rise in flow rate was noted once the excipients were included, with values of 3.030 g/s for F3 and 2.673 g/s for F4 recorded when passing the powders through the 15 mm nozzle without stirring. The proper choice of excipients for both formulations resulted in compound powders with appropriate dynamic characteristics for the capsule-filling operation. Nevertheless, F4, which is based on resveratrol, shows a lower flowability than F3, which was anticipated given the results of the initial powders (Table 3).

### 2.6. Antioxidant Properties of the Capsules’ Content

#### 2.6.1. DPPH Radical Scavenging Activity

**Figure 6:**
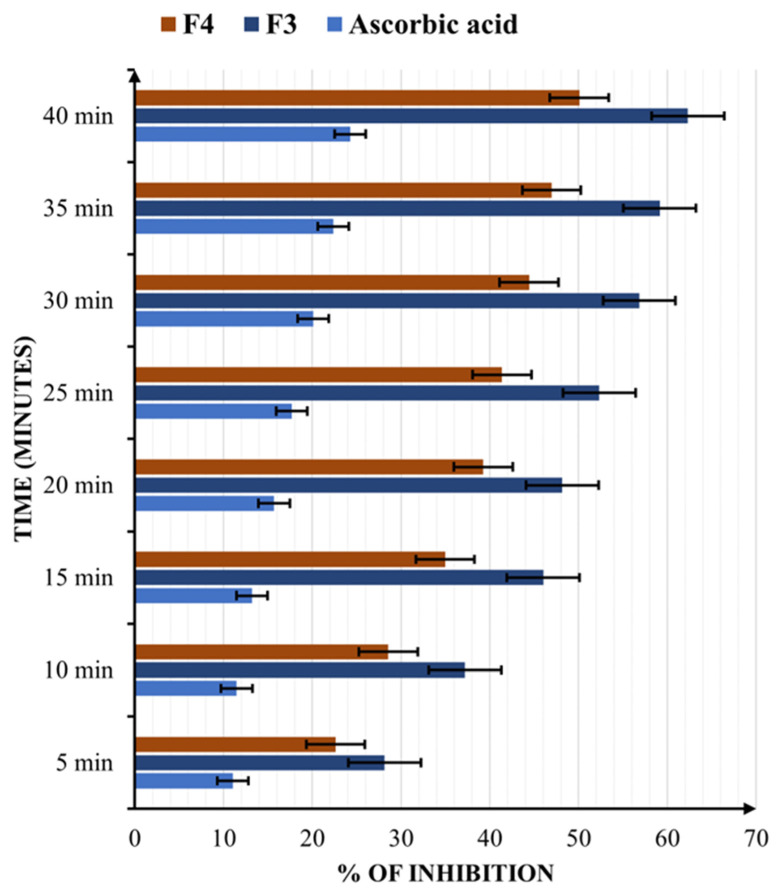
The DPPH radical scavenging activities of the final formulations: 2.89 mg/mL for F3 (1.03 mg/mL of quercetin, 0.08 mg/mL of biotin, and 1.03 mg/mL of coenzyme Q10) and 3.39 mg/mL for F4 (1.21 mg/mL of resveratrol, 0.09 mg/mL of biotin, and 1.21 mg/mL of coenzyme Q10) and 4.93 mg/mL of ascorbic acid. Results are indicated as mean ± SD of data (n = 5).

The percentage of DPPH radical inhibition registered by the samples and the positive control, vitamin C (4.93 mg/mL), are shown in Figure 6.

Compared to the reference used, ascorbic acid, which has been shown to have potent antioxidative properties, both studied formulations were found to be more active. Still, F3 turned out to be more efficient (62.3% after 40 min) than F4 (50.08% at 40 min). The results show that quercetin has a stronger electron-donating ability than resveratrol, but the difference between F3 and F4 is not remarkable. Nevertheless, both formulations exhibit superior antioxidant performance compared to vitamin C.

#### 2.6.2. ABTS Radical Scavenging Activity

**Figure 7:**
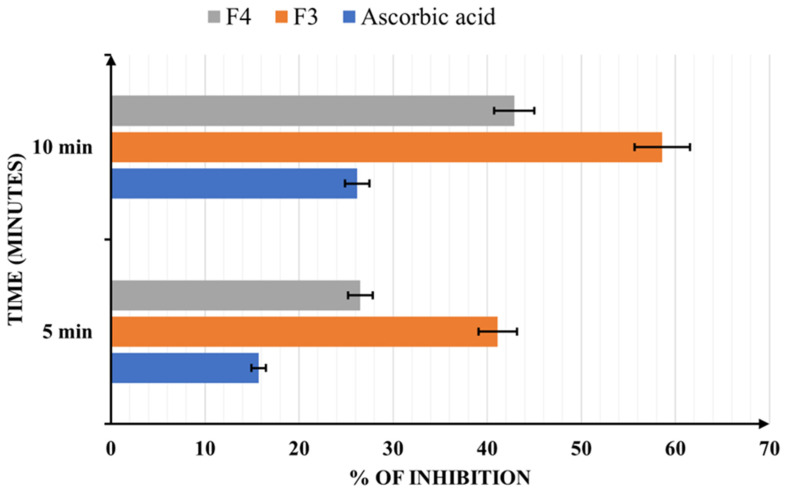
The ABTS radical scavenging activity of the final formulations: 2.89 mg/mL for F3 (1.03 of mg/mL quercetin, 0.08 mg/mL of biotin, and 1.03 mg/mL of coenzyme Q10) and 3.39 mg/mL for F4 (1.21 mg/mL of resveratrol, 0.09 mg/mL of biotin, and 1.21 mg/mL of coenzyme Q10) and 4.93 mg/mL of ascorbic acid. Results are indicated as mean ± SD of data (n = 5).

Figure 7 presents the ABTS radical scavenging activity of the samples.

The results for ABTS scavenging are similar to the previous analysis, with F3 showing the highest antioxidant efficacy (48.62% after 10 min). However, the data demonstrate that both samples are more effective in donating electrons than ascorbic acid 2.8 mM, which was only 26.19% at 10 min.

#### 2.6.3. FRAP Assay

**Figure 8:**
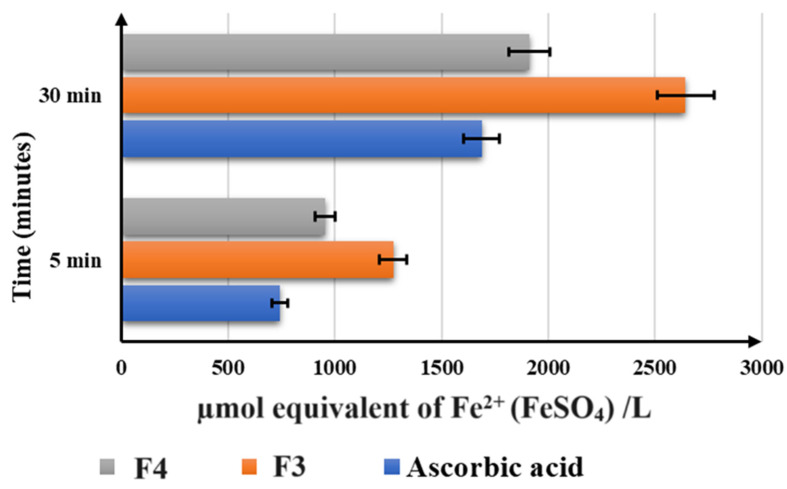
The FRAP assay results of the final formulations: 2.89 mg/mL for F3 (1.03 mg/mL of quercetin, 0.08 mg/mL of biotin, and 1.03 mg/mL of coenzyme Q10) and 3.39 mg/mL for F4 (1.21 mg/mL of resveratrol, 0.09 mg/mL of biotin, and 1.21 mg/mL of coenzyme Q10) and 4.93 mg/mL of ascorbic acid. Results are indicated as mean ± SD of data (n = 5).

Figure 8 presents the FRAP assay results (µmol equivalent of Fe^2+^ (FeSO_4_)/L) recorded at 5 and 30 min.

Regarding the antioxidant capacity of the formulations, the results of the FRAP study are in agreement with the outcomes of ABTS and DPPH analysis. F3 was shown to be the most efficient antioxidant, followed by F4, which has a higher reducing activity than the reference.

### 2.7. Final Capsules’ Qualitative Evaluation

Table 4 displays the results of the evaluation of the qualitative properties of the manufactured capsules.

The two batches exhibit proper mass uniformity according to the enforced guidelines, with a minor variation between them (347.18 mg for F3 and 348.02 mg for F4). Both formulations proved to be fast-disintegrating, with no significant difference amongst them. Still, F3 disintegrated more rapidly in the aqueous medium (419 s) than F4 (487 s), revealing the influence of quercetin and resveratrol on the disintegration performance of the final product.

## 3. Discussion

The presence of larger particles in F2 and F4 compared to F1 and F3 is certainly due to the inclusion of resveratrol in the formulations. Still, it can also be explained by the higher amount of moisture found in F2 and F4. The humidity in powders increases the cohesion between particles by forming liquid bonds [67], which leads to a growth in particle sizes due to the formation of agglomerates. Sun C.C. [68] discovered differences in pharmacotechnical properties of microcrystalline cellulose in the moisture range of 3–5% but decided that this range is optimal for suitable powder flowability. On the other hand, Khan et al. [69] considered that humidity over 3% breaks the particle bonds in a compact arrangement, as in capsule filling. Also, Koumbogle et al. [70] revealed that moisture evaporates from a compacted powder bed to the surrounding environment, proving the importance of maintaining a low humidity content of the materials for solid-dosage products. The final formulations of the filling powders for capsules displayed an adequate water content in comparison to the initial active ingredients’ mixtures conforming to the proper selection of the excipients.

The active ingredients have a major impact on the volumetric properties of the materials, but the addition of excipients led to a noticeable improvement in the CI and HR parameters. According to Chaerunisa et al. [71], microcrystalline cellulose has a porous structure, which accounts for its relatively low bulk density. As a result, F3 and F4 exhibited reduced bulk and tapped densities compared to F1 and F2. When quercetin is incorporated into the mixture (F1), both bulk densities increase. Still, the values of CI and HR do not exhibit adequate flow or compression properties, which can be explained by the small size of the particles. Janssen et al. [72] stated that small particles have poor flowability because they normally move slowly. Instead, CI and HR decrease remarkably after the addition of excipients, and F3 has the proper characteristics for encapsulation powders. Although F2 has much larger particles, its density is considerably lower than that of F1, and CI and HR values are superior. This behaviour is justified by the higher moisture content of the samples, which is due to the influence of resveratrol. Even though the excipients used significantly reduced the humidity amount, the volumetric properties for F4 revealed weaker mechanical attributes compared to F3. The results are in agreement with Zhenfeng et al.’s findings [73], which demonstrated that the relative humidity of the powders strongly affects the flowability and filling performance of the materials. Resveratrol appears to noticeably reduce the flowability of the materials; even if its particles are larger than those of quercetin, its high moisture content basically enhances the cohesiveness of the powders by creating liquid bonds between particles. The selected excipients notably optimise the mechanical properties of the two active ingredient mixtures, proving an effective formulation of the capsule contents through an accurate selection of the types and quantities of excipients. The filler, lubricant, and even superdisintegrant excipients significantly enhanced the poor flowability of the active ingredients’ mixtures. Sodium starch glycolate was previously reported to possess good flowing qualities [74]. The present results confirm the numerous reports [75,76,77,78,79] on the ability of microcrystalline cellulose to improve the powders’ flowability. To produce appropriate and uniform capsules, it is crucial to optimise the flow ability of the active components throughout the filling process. Stranzinger et al. [80] evidenced that weight fluctuation, fill weight, and layer uniformity are highly dependent on the mechanical properties of the powder. The current investigation showed that F1 and F2 had poor flowability, which was difficult to remedy given the high concentrations of active ingredients in the formulations. This prevented the use of large amounts of excipients as patients would not accept larger capsules. It was a challenge to solve this constraint by utilizing only small amounts of a limited number of excipients, making the excipient selection process a crucial step of the formulation.

The observed superior antioxidant activity of the studied formulations, F3 and F4, compared to the reference ascorbic acid (vitamin C), underscores the potential efficacy of the novel antioxidant blends in combating oxidative stress [81]. The findings suggest that the combined action of quercetin, biotin, coenzyme Q10, and resveratrol within the formulations contributes to their enhanced antioxidant performance, surpassing that of individual antioxidants such as vitamin C. The synergistic effect observed in F3 and F4 formulations surpasses the antioxidant performance of individual components, enhancing their overall antioxidant capacity.

Quercetin, biotin, coenzyme Q10, and resveratrol each possess distinct antioxidant mechanisms and molecular targets within the cellular antioxidant defence system. Quercetin, a flavonoid abundant in fruits and vegetables, is known for its potent antioxidant properties, attributed to its ability to donate electrons and scavenge free. Numerous studies have highlighted quercetin’s role in protecting against oxidative stress-induced damage and its potential therapeutic benefits in neurodegenerative disease radicals [82]. Similarly, biotin, an essential B vitamin, has been shown to enhance antioxidant defences and promote mitochondrial function, thereby mitigating oxidative damage [83]. Coenzyme Q10, a key component of the electron transport chain, acts as a potent lipid-soluble antioxidant, protecting cell membranes from oxidative damage and supporting cellular energy production [84,85]. Resveratrol, found in red grapes and berries, possesses antioxidant, anti-inflammatory, and neuroprotective properties, attributed to its activation of sirtuins and modulation of oxidative stress response pathways [86]. Individually, these antioxidants have demonstrated significant antioxidant activity in various studies [87,88]. The observed differences in antioxidant performance between F3 and F4 may be attributed to variations in the concentrations of individual ingredients or their interactions within the formulations. The superior antioxidant activity of F3 and F4, as demonstrated by ABTS, DPPH, and FRAP assays, highlights their potential as effective interventions against oxidative stress-related conditions. The results support the rationale for combining multiple antioxidants with complementary mechanisms of action to achieve optimal antioxidant effects. For example, quercetin may enhance the bioavailability and cellular uptake of coenzyme Q10, thereby augmenting mitochondrial antioxidant capacity [89]. Similarly, resveratrol may potentiate the antioxidant activity of biotin by modulating intracellular signaling pathways involved in oxidative stress response [90]. Our study aligns with previous research indicating that combinations of natural antioxidants can exhibit synergistic effects, resulting in enhanced antioxidant activity compared to individual compounds. For example, studies have demonstrated synergism between quercetin and resveratrol in scavenging free radicals and protecting against oxidative stress-induced cellular damage [91,92,93]. Similarly, the combination of coenzyme Q10 and resveratrol has been shown to exert synergistic effects in improving mitochondrial function and reducing oxidative stress in animal models [94,95]. These findings support the notion that the combined action of quercetin, biotin, coenzyme Q10, and resveratrol in F3 and F4 formulations may contribute to their superior antioxidant performance.

Our discussion of the potential mechanisms underlying the synergistic antioxidant effects of F3 and F4 formulations is supported by existing literature on the individual bioactive compounds. For instance, quercetin has been shown to enhance the expression and activity of antioxidant enzymes such as superoxide dismutase (SOD) and catalase (CAT) through activation of the nuclear factor erythroid 2-related factor 2 (Nrf2) signaling pathway [96]. Biotin, in turn, has been reported to enhance mitochondrial function and reduce oxidative stress by promoting the activity of biotin-dependent carboxylases involved in energy metabolism [97]. Coenzyme Q10, as a crucial component of the mitochondrial electron transport chain, plays a central role in cellular energy production and antioxidant defence [98]. Resveratrol, on the other hand, exerts antioxidant effects through multiple mechanisms, including activation of sirtuin proteins, inhibition of pro-inflammatory signaling pathways, and modulation of oxidative stress-responsive genes [99]. By integrating these mechanistic insights, the present study provides a comprehensive understanding of how the combined action of quercetin, biotin, coenzyme Q10, and resveratrol within F3 and F4 formulations may confer robust antioxidant protection against oxidative stress. Existing clinical studies have provided evidence supporting the beneficial effects of individual antioxidants such as coenzyme Q10 and resveratrol in improving markers of oxidative stress and neurodegeneration in patients with Parkinson’s disease and Alzheimer’s disease [100,101,102].

The correlation between mass variations and mechanical attributes of powders has been demonstrated in several studies. Cronin et al. [103] demonstrated the relationship between powder size dispersion, materials density, and mass uniformity. Rowe et al. [104] also found that the main physical cause of the weight variability is the flowability of the powders, which is influenced by the involvement of the multiple cohesive forces between particles. The results on the mass uniformity of the capsules proved the appropriate pharmacotechnical properties of the filling materials with adequate flowability and compressibility as shown in the formulation studies.

There was a slight variation between the formulations in terms of the disintegration properties. The appropriate choice of excipients in the formulations is validated by the proper disintegration times exhibited by both batches in compliance with European Pharmacopoeia criteria. However, the amount of the superdisintegrant utilised in both formulations, sodium starch glycolate, is the same, suggesting that it is not the only agent that promotes the disintegration of the particles [105,106]. In addition, the contents of the excipients are the same, indicating that the active components significantly affect the disintegration performance. It is confirmed that the quercetin included in the F3 blend offers superior disintegration capacity compared to the resveratrol contained by F4.

In light of the encouraging results obtained from our in vitro antioxidant assays, we are motivated to explore the antioxidant efficacy of the studied mixtures in biological systems. Specifically, we plan to conduct cell-line-based assays to validate and further elucidate the observed antioxidant activity. By investigating the performance of our mixtures in more physiologically relevant contexts, we aim to provide a comprehensive understanding of their potential applications in combating oxidative stress-related conditions.

## 4. Materials and Methods

### 4.1. Materials

Quercetin and resveratrol (Shanghai Zhongxin Yuxiang Chemical Co., Ltd., Shanghai, China), biotin (Zheijiang Shengda Bio-Pharm Co., Ltd., Taizhou, China), and coenzyme Q10 (Changsha Phyto Nutrition Inc., Changsha, China) were purchased from Fagron Hellas, Trikala, Greece. All compounds were used as received. Avicel PH 102 was provided by DuPont^™^ Nutrition and Health, Newark, DE, USA, EXPLOTAB^®^ by JRS PHARMA GmbH & Co. KG, Rosenberg, Germany, and LIGAMED^®^ MF-2-V by Peter Graven NV, Venlo, Limburg, The Netherlands. ABTS (2,2′-azino-bis(3-ethylbenzothiazoline-6-sulfonic acid), DPPH (2,2-Diphenyl-1-picrylhydrazyl), and TPTZ (2,4,6-tri(2-pyridyl)-1,3,5-triazine) were provided by Sigma-Aldrich Chemie GmbH, Taufkirchen, Germany.

### 4.2. Methods

#### 4.2.1. Methods of Physical–Chemical Analysis

The Fourier Transform Infrared Spectra (FTIR) were recorded in the 4000–400 cm^−1^ domain using a Nicolet 6700 instrument (Thermo Electron Corporation, Waltham, MA, USA), in KBr pellets, in transmittance mode with a sensitivity of 4 cm^−1^. X-ray diffraction analysis was carried out using a PANalytical Empyrean diffractometer (Thermo Fisher, Waltham, MA, USA), at room temperature, using a Cu X-ray tube (λ Cu Kα1 = 1.541874 Ǻ), in the range of 20–80° at a 0.02° scan step in a Bragg–Brentano geometry. Thermal analysis (TG/DTG) was accomplished with a Mettler Toledo TGA/SDTA851e instrument (Mettler-Toledo GmbH, Greifensee, Switzerland). The experiments were recorded in air atmosphere at a flow rate of 80 mL min^−1^ with 10 °C/min heating rate. The SEM images were recorded using scanning electron microscopy (SEM) (Thermo Fisher Scientific, GmbH, Dreieich, Germany) with a Quanta 3D field emission in high vacuum mode, at a 2 kV accelerating voltage.

#### 4.2.2. Preformulation Analysis

It is essential to determine the pharmacotechnical attributes of the active ingredient mixtures in order to produce acceptable capsule formulations. The precise selection of the required types and quantities of excipients will be aided by knowledge of these properties. Two mixtures of active ingredients were proposed and studied: F1, which contains quercetin: biotin: coenzyme Q10 in a mass ratio of 25:1:25, and F2, which includes resveratrol: biotin: coenzyme Q10 in the same mass ratio of 25:1:25.

*Particle size distribution (PSD)* was assessed using a CISA Sieve Shaker Mod. RP 10 manufactured by CisaCedaceria Industrial, Barcelona, Spain, using 45 g of each formulation. The samples were positioned on the sieves, which are stacked progressively from top to bottom with a mesh of progressively smaller sizes. The materials that remained on each sieve after the setting was shaken for 20 min were collected and weighed.

*Flowability*: In order to measure the angle of repose, and the time and rate of flowing, 50 g samples of each formulation were passed through an orifice with a predetermined diameter. Pharma Test Apparatebau GmbH, Hainburg, Germany’s Automated Powder and Granulate Testing System PTG-S3, was used for the analysis.

*Compactness* was determined using a Vankel Tap Density Tester, produced by Vankel Industries Inc., Cary, NC, USA. The bulk and tapped densities, the Hausner ratio (*HR*), and the Carr Index (*CI*) were all calculated. The bulk volume is the volume occupied by 50 g of each powder. The materials were then mechanically tapped a specified number of times before the tapped volume was measured. Equation (1) for determining *CI* and the ratio of tapped density to bulk density, or *HR*, is used for the evaluation of powders’ compressibility behaviour [107]:

(1) $$CI\left(\%\right)=100\times \frac{\left(\rho_{tapped}-\rho_{bulk}\right)}{\rho_{tapped}}$$

where ρ_tapped_ is the tapped density (kg/m^3^), and ρ_bulk_ is the bulk density (kg/m^3^).

An HR value of less than 1.25 points out that the powder has a free flow, and a CI value below 10 indicates that the powder has excellent flowability and compactness.

*Moisture content* was measured as the loss on drying using the thermogravimetric method and a Mettler-Toledo GmbH HR 73 halogen humidity analyser at Greifensee, Switzerland [108].

#### 4.2.3. Formulation and Preparation of the Filling Powders for Capsules

The selection of the appropriate amounts and types of excipients becomes a crucial phase in the formulation process when taking into account the outcomes of the preformulation tests and the maximum mass permitted for the final capsules. The biopharmaceutical performance of the product will be imprinted by its processibility and quality, both of which depend critically on the formulation of the capsules’ contents. Maintaining a manageable moderate mass was another crucial consideration because the goal was to produce capsules that could be easily swallowed and accepted by patients. The amount per dosage unit was decided based on the efficacy of the active ingredients that already have been demonstrated and published [109]. The studied formulations are shown in Table 5.

The preformulation studies revealed the poor flowability of the active ingredient mixtures; thus, a filler with great rheological characteristics was required. The microcrystalline cellulose PH 102 was discovered to be the best choice for this. The sodium starch glycolate’s function is to provide a shorter disintegration, but it also has excellent flowing properties. Due to its large specific surface area, which would improve the powder flowability, Ligamed^®^ MF-2-V was selected as the lubricant. All ingredients were passed through a 20-mesh, weighed, and combined in a CMP 12 Plexiglas cube mixer made by Pharmag GmbH in Kliphausen, Germany, and then mixed at 35 rpm for 20 min at room temperature.

#### 4.2.4. Capsules’ Content Evaluation

Using the same techniques as previously, the resulting materials’ pharmacotechnical properties were tested, including their fineness, humidity content, flow, and compactness features. Also, their antioxidant activity was determined. Numerous investigations on the antioxidant qualities of the substances chosen for the study are presented in the literature, but none of them was performed on the final mixtures, which also contain excipients. To determine whether the antioxidant ability is maintained in the suggested combinations, it was decided to conduct these analyses on the filling complex powders for the capsules. Three techniques (ABTS, DPPH, and FRAP) were utilised to measure the antioxidant activity, and the findings were compared to acid ascorbic, which was employed as a positive control [59,107]. The decision to use a higher concentration of ascorbic acid (2.8 mM/4.93 mg/mL) in our experiments was thoughtfully analysed. Ascorbic acid is a potent antioxidant with well-established radical scavenging capabilities. As previous research [107] has shown varying concentrations of ascorbic acid to be effective, ranging from 87.5 μM to 2.8 mM, we aimed to explore the dose–response relationship and evaluate the antioxidant activity of our mixtures across a higher concentration of ascorbic acid. By employing this concentration, we sought to discern whether our formulations could surpass the antioxidative potential of ascorbic acid, providing valuable insights into their utility in practical applications requiring robust antioxidant activity. The results regarding the antioxidant activity are expressed as the mean ± standard error of the mean (n = 5). The presentation of results utilised arithmetic means and standard deviations to describe central tendency and variability, respectively, using the Excel program—Version 2404. Error bars were added to graphical representations of the data to depict the variability associated with each data point visually. Error bars were calculated based on either standard deviation or standard error, as appropriate for each dataset.

*ABTS radical scavenging activity*

A method that has been previously described was used to evaluate the total antioxidant capacity [110]. The interaction between ABTS and potassium persulfate took place for 12 h at room temperature and in complete darkness to produce the cationic radical ABTS^●+^. At 734 nm, the reagent’s absorbance was 0.70 ± 0.02. Following the addition of the material, the decrease in absorbance was observed for 1 min at 734 nm, and the values were calculated using a Trolox-created calibration curve. The results were presented as mmol Trolox equivalents/mg formulation.

*DPPH radical scavenging activity*

The Margina et al. [111] approach was used to carry it out. Every five minutes, for 40 min, the absorbances of the samples and DPPH solution were measured at 505 nm. After 40 min, the findings are shown as a percentage of the optical density’s decrease from the starting point.

Radical scavenging activity (*RSA*%), a measure of the DPPH scavenging action, was computed using Equation (2):

(2) $$RSA\%=\frac{\left(Abs_{0}-Abs_{p}\right)}{Abs_{0}}\times 100$$

where Abs_0_ is the absorbance of DPPH solution (reference) at *t*_0_ (initial optical density); Abs_p_ is the absorbance of the samples measured at different periods of times *t* (sample optical density).

*FRAP assay*

The Nair et al. [112] method was used to determine the FRAP assay. At 5 and 30 min following preparation, the samples that had been combined with FRAP solution had their absorbances measured at 593 nm. Fe^2+^ calibration curves in methanol were used to calculate the FRAP values (µmol equivalent of Fe^2+^ (FeSO_4_)/L).

#### 4.2.5. Final Capsules’ Manufacturing and Assessment

Since each hard gelatin “1” size capsule can store around 400 mg of powder, white-coloured hard gelatin capsules were employed as the product’s shells. Using a FagronLabTM FG1 semi-automatic encapsulation apparatus made by Gako Konietzko GmbH in Bamberg, Germany, they were filled with content. Specific quality control procedures were used for the finished two-piece capsules.

*Mass uniformity*

It was assessed in accordance with the European Pharmacopoeia [66] guidelines by weighing 10 filled capsules of each formulation separately and then just the empty shells. The mean value of the filling mass that contains the active substances is used to calculate and express the mass difference.

*In vitro disintegration time*

It was carried out using the Erweka DT 3 apparatus, manufactured by Erweka^®^ GmbH, Langen, Germany, following compendial requirements [66,113]. Six tablets of each formulation were tested in distilled water at 37 ± 0.5 °C. The time needed for complete disintegration, leaving nothing on the screen, but a few fragments of the capsule’s shell, was recorded.

## 5. Conclusions

In conclusion, our study demonstrates the superior antioxidant activity of novel formulations F3 and F4 compared to the reference antioxidant, ascorbic acid (vitamin C). The observed efficacy of F3 and F4 formulations in combating oxidative stress underscores their potential as therapeutic agents for mitigating oxidative damage and associated pathologies. The combined action of quercetin, biotin, coenzyme Q10, and resveratrol within F3 and F4 formulations contributes to their enhanced antioxidant performance, surpassing that of individual antioxidants such as vitamin C. This synergistic effect is supported by mechanistic insights into the diverse antioxidant mechanisms and molecular targets of the bioactive compounds. Our findings have significant implications for the development of antioxidant-based therapies targeting oxidative stress-related disorders. By harnessing the synergistic interactions among quercetin, biotin, coenzyme Q10, and resveratrol, F3 and F4 formulations offer a multifaceted approach to bolstering the body’s antioxidant defence system and mitigating the detrimental effects of oxidative stress on cellular function and health. Future studies should focus on elucidating the underlying mechanisms of action, optimizing formulation compositions, and evaluating the long-term safety and efficacy of F3 and F4 formulations in preclinical and clinical settings. Comparative studies with existing antioxidant therapies will further validate the superiority of F3 and F4 formulations and guide their translation into clinical practice. Overall, our findings contribute to advancing the field of antioxidant research and provide a basis for the development of novel therapeutic interventions aimed at combating oxidative stress and improving health outcomes in patients at risk of neurodegenerative diseases and other oxidative stress-related disorders.

## Figures and Tables

**Table 1:** Thermal characteristics from TG/DTG curves of studied samples.

Compound	1st Step	2nd Step	3rd Step	T °C/Residue
Quercetin	20–100 °C T_DTG_ = 93.5 °C Mass loss = 4.8%	220–395 °C T_DTG_ = 272.7 °C Mass loss = 39.2%	395–500 °C Mass loss = 22.5%	500 °C/16.7%
Biotin	-	200–390 °C T_DTG_ = 331.5 °C T_DTG_ = 365.6 °C Mass loss = 42.2%	350–480 °C T_DTG_ = 448 °C Mass loss = 32.8%	500 °C/25%
Coenzyme Q10	-	240–350 °C T_DTG_ = 320.8 °C Mass loss = 65.8%	350–500 °C T_DTG_ = 388 °C Mass loss = 26.23%	500 °C/7.97%
Resveratrol	20–100 °C T_DTG_ = 51.3 °C Mass loss = 4.7%	205–350 °C T_DTG_ = 209.8 °C T_DTG_ = 320.6 °C Mass loss = 36.8%	350–500 °C T_DTG_ = 490.8 °C Mass loss = 50%	500 °C/8.5%
F1	20–140 °C Mass loss = 6.8%	190–380 °C T_DTG_ = 300.2 °C T_DTA_ = 305 °C Shoulder T_DTG_ = 340.5 °C Mass loss = 45.7%	380–500 °C T_DTG_ = 479.8 °C T_DTA_ = 482.9 °C Mass loss = 40.05%	500 °C/7.45%
F2	20–100 °C Mass loss = 3.8%	180–414 °C T_DTG_ = 318 °C T_DTA_ = 334 °C Mass loss = 57%	414–500 °C T_DTG_ = 488 °C T_DTA_ = 494 °C Mass loss = 29.4%	500 °C/9.8%
F3	20–140 °C T_DTG_ = 111 °C T_DTA_ = 118 °C Mass loss = 6.1%	175–367 °C T_DTG_ = 298 °C T_DTA_ = 300 °C Mass loss = 40.4%	367–500 °C T_DTG_ = 485 °C T_DTA_ = 491 °C Mass loss = 41.8%	500 °C/11.7%
F4	20–120 °C Mass loss = 2.7%	120–400 °C T_DTG_ = 232 °C T_DTG_ = 319.5 °C T_DTA_ = 332 °C Mass loss = 51.1%	400–500 °C T_DTG_ = 490 °C T_DTA_ = 496.6 °C Mass loss = 33.2%	500 °C/13%

**Table 2:** Volumetric properties of the materials.

Parameter *	F1	F2	F3	F4
Bulk density (g/mL)	0.638 ± 0.35	0.357 ± 0.22	0.611 ± 0.08	0.330 ± 0.51
Tapped density (g/mL)	0.845 ± 0.24	0.577 ± 0.17	0.758 ± 0.11	0.429 ± 0.32
Carr Index (CI) (%)	24.497	38.128	19.393	23.076
Hausner’s ratio (HR)	1.324	1.617	1.24	1.30

* mean ± SD.

**Table 3:** Flowing parameters of the powders.

Parameter *	F1	F2	F3	F4
Flow time (s)	23.6 ± 1.39	27.2 ± 2.72	16.5 ± 1.66	18.7 ± 1.94
Angle of repose (θ°)	41.34 ± 2.58	46.12 ± 2.76	27.85 ± 2.12	28.34 ± 1.97
Flow rate (g/s)	2.118	1.838	3.030	2.673

* mean ± SD.

**Table 4:** Qualitative properties of the capsules.

Parameters *	Formulation Code	
	F3	F4
Mass uniformity (mg)	347.18 ± 1.54	348.02 ± 2.61
In vitro disintegration time (s)	419 ± 3.0	487 ± 2.0

* mean ± SD.

**Table 5:** The capsules’ content compositions.

Ingredients	Quantity (mg)/Capsule		Role in Formulation
	F3	F4	
Quercetin	125	-	Active ingredient
Biotin	10	10	Active ingredient
Coenzyme Q10	125	125	Active ingredient
Resveratrol	-	125	Active ingredient
Avicel PH 102—Microcrystalline cellulose	79.5	79.5	Filler
Explotab^®^—Sodium starch glycolate	7	7	Superdisintegrant
Ligamed^®^ MF-2-V—Magnesium stearate	3.5	3.5	Lubricant
Total	350	350	

## Data Availability

Data is contained within the article.

## References

[1] Bhattacharyya A., Chattopadhyay R., Mitra S., Crowe S.E. (2014). Oxidative stress: An essential factor in the pathogenesis of gastrointestinal mucosal diseases. Physiol. Rev..

[2] Xie X., He Z., Chen N., Tang Z., Wang Q., Cai Y. (2019). The Roles of Environmental Factors in Regulation of Oxidative Stress in Plant. Biomed. Res. Int..

[3] Aranda-Rivera A.K., Cruz-Gregorio A., Arancibia-Hernández Y.L., Hernández-Cruz E.Y., Pedraza-Chaverri J. (2022). RONS and Oxidative Stress: An Overview of Basic Concepts. Oxygen.

[4] Sies H., Berndt C., Jones D.P. (2017). Oxidative Stress. Ann. Rev. Biochem..

[5] Zheng F., Gonçalves F.M., Abiko Y., Li H., Kumagai Y., Aschner M. (2020). Redox toxicology of environmental chemicals causing oxidative stress. Redox Biol..

[6] Rahal A., Kumar A., Singh V., Yadav B., Tiwari R., Chakraborty S., Dhama K. (2014). Oxidative stress, prooxidants, and antioxidants: The interplay. Biomed. Res. Int..

[7] Aseervatham G.S., Sivasudha T., Jeyadevi R., Arul Ananth D. (2013). Environmental factors and unhealthy lifestyle influence oxidative stress in humans--an overview. Environ. Sci. Pollut. Res. Int..

[8] Pham-Huy L.A., He H., Pham-Huy C. (2008). Free radicals, antioxidants in disease and health. Int. J. Biomed. Sci..

[9] Enkavi G., Javanainen M., Kulig W., Róg T., Vattulainen I. (2019). Multiscale Simulations of Biological Membranes: The Challenge to Understand Biological Phenomena in a Living Substance. Chem. Rev..

[10] Pizzino G., Irrera N., Cucinotta M., Pallio G., Mannino F., Arcoraci V., Squadrito F., Altavilla D., Bitto A. (2017). Oxidative Stress: Harms and Benefits for Human Health. Oxid. Med. Cell Longev..

[11] Hajam Y.A., Rani R., Ganie S.Y., Sheikh T.A., Javaid D., Qadri S.S., Pramodh S., Alsulimani A., Alkhanani M.F., Harakeh S. (2022). Oxidative Stress in Human Pathology and Aging: Molecular Mechanisms and Perspectives. Cells.

[12] Cervantes Gracia K., Llanas-Cornejo D., Husi H. (2017). CVD and Oxidative Stress. J. Clin. Med..

[13] Vona R., Pallotta L., Cappelletti M., Severi C., Matarrese P. (2021). The Impact of Oxidative Stress in Human Pathology: Focus on Gastrointestinal Disorders. Antioxidants.

[14] Inokuma T., Haraguchi M., Fujita F., Tajima Y., Kanematsu T. (2009). Oxidative stress and tumor progression in colorectal cancer. Hepatogastroenterology.

[15] Reuter S., Gupta S.C., Chaturvedi M.M., Aggarwal B.B. (2010). Oxidative stress, inflammation, and cancer: How are they linked?. Free Radic. Biol. Med..

[16] Klaunig J.E. (2018). Oxidative Stress and Cancer. Curr. Pharm. Des..

[17] Violi F., Loffredo L., Carnevale R., Pignatelli P., Pastori D. (2017). Atherothrombosis and Oxidative Stress: Mechanisms and Management in Elderly. Antioxid. Redox Signal..

[18] Chen X., Guo C., Kong J. (2012). Oxidative stress in neurodegenerative diseases. Neural Regen. Res..

[19] Olufunmilayo E.O., Gerke-Duncan M.B., Holsinger R.M.D. (2023). Oxidative Stress and Antioxidants in Neurodegenerative Disorders. Antioxidants.

[20] Hantikainen E., Trolle Lagerros Y., Ye W., Serafini M., Adami H.O., Bellocco R., Bonn S. (2021). Dietary Antioxidants and the Risk of Parkinson Disease: The Swedish National March Cohort. Neurology.

[21] Frank B., Gupta S. (2005). A review of antioxidants and Alzheimer’s disease. Ann. Clin. Psychiatry.

[22] Elangovan S., Holsinger R.M.D. (2020). Cyclical amyloid beta-astrocyte activity induces oxidative stress in Alzheimer’s disease. Biochimie.

[23] Marzola P., Melzer T., Pavesi E., Gil-Mohapel J., Brocardo P.S. (2023). Exploring the Role of Neuroplasticity in Development, Aging, and Neurodegeneration. Brain Sci..

[24] Li M., Fu X., Xie W., Guo W., Li B., Cui R., Yang W. (2020). Effect of Early Life Stress on the Epigenetic Profiles in Depression. Front. Cell Dev. Biol..

[25] Knopman D.S., Amieva H., Petersen R.C., Chételat G., Holtzman D.M., Hyman B.T., Nixon R.A., Jones D.T. (2021). Alzheimer Disease. Nat. Rev. Dis. Prim..

[26] Nayak M., Das D., Pradhan J., Ahmed R.G., Laureano-Melo R., Dandapat J. (2022). Epigenetic signature in neural plasticity: The journey so far and journey ahead. Heliyon.

[27] Wu A., Ying Z., Gomez-Pinilla F. (2004). The interplay between oxidative stress and brain-derived neurotrophic factor modulates the outcome of a saturated fat diet on synaptic plasticity and cognition. Eur. J. Neurosci..

[28] Shohag S., Akhter S., Islam S., Sarker T., Sifat M.K., Rahman M.M., Islam M.R., Sharma R. (2022). Perspectives on the Molecular Mediators of Oxidative Stress and Antioxidant Strategies in the Context of Neuroprotection and Neurolongevity: An Extensive Review. Oxid. Med. Cell Longev..

[29] Plascencia-Villa G., Perry G. (2023). Roles of Oxidative Stress in Synaptic Dysfunction and Neuronal Cell Death in Alzheimer’s Disease. Antioxidants.

[30] Toricelli M., Pereira A.A.R., Souza Abrao G., Malerba H.N., Maia J., Buck H.S., Viel T.A. (2021). Mechanisms of neuroplasticity and brain degeneration: Strategies for protection during the aging process. Neural Regen. Res..

[31] Maharjan R., Diaz Bustamante L., Ghattas K.N., Ilyas S., Al-Refai R., Khan S. (2020). Role of Lifestyle in Neuroplasticity and Neurogenesis in an Aging Brain. Cureus.

[32] Jackson M., Marks L., May G.H.W., Wilson J.B. (2018). The genetic basis of disease. Essays Biochem..

[33] Shadfar S., Parakh S., Jamali M.S., Atkin J.D. (2023). Redox dysregulation as a driver for DNA damage and its relationship to neurodegenerative diseases. Transl. Neurodegener..

[34] Kushwah N., Bora K., Maurya M., Pavlovich M.C., Chen J. (2023). Oxidative Stress and Antioxidants in Age-Related Macular Degeneration. Antioxidants.

[35] Kok J.R., Palminha N.M., Dos Santos Souza C., El-Khamisy S.F., Ferraiuolo L. (2021). DNA damage as a mechanism of neurodegeneration in ALS and a contributor to astrocyte toxicity. Cell Mol. Life Sci..

[36] Sun Y., Curle A.J., Haider A.M., Balmus G. (2020). The role of DNA damage response in amyotrophic lateral sclerosis. Essays Biochem..

[37] Madabhushi R., Pan L., Tsai L.H. (2014). DNA damage and its links to neurodegeneration. Neuron.

[38] Wilhelm T., Said M., Naim V. (2020). DNA Replication Stress and Chromosomal Instability: Dangerous Liaisons. Genes.

[39] Popovici V., Matei E., Cozaru G.-C., Bucur L., Gîrd C.E., Schröder V., Ozon E.A., Sarbu I., Musuc A.M., Atkinson I. (2022). Formulation and Development of Bioadhesive Oral Films Containing *Usnea barbata* (L.) F.H.Wigg Dry Ethanol Extract (F-UBE-HPC) with Antimicrobial and Anticancer Properties for Potential Use in Oral Cancer Complementary Therapy. Pharmaceutics.

[40] Popovici V., Matei E., Cozaru G.C., Bucur L., Gîrd C.E., Schröder V., Ozon E.A., Karampelas O., Musuc A.M., Atkinson I. (2022). Evaluation of *Usnea barbata* (L.) Weber ex F.H. Wigg Extract in Canola Oil Loaded in Bioadhesive Oral Films for Potential Applications in Oral Cavity Infections and Malignancy. Antioxidants.

[41] Vassalle C., Maltinti M., Sabatino L. (2020). Targeting Oxidative Stress for Disease Prevention and Therapy: Where Do We Stand, and Where Do We Go from Here. Molecules.

[42] Engedal N., Žerovnik E., Rudov A., Galli F., Olivieri F., Procopio A.D., Rippo M.R., Monsurrò V., Betti M., Albertini M.C. (2018). From Oxidative Stress Damage to Pathways, Networks, and Autophagy via MicroRNAs. Oxid. Med. Cell. Longev..

[43] Fioravanti A., Pirtoli L., Giordano A., Dotta F. (2020). Crosstalk between MicroRNA and Oxidative Stress in Physiology and Pathology. Int. J. Mol. Sci..

[44] Tan B.L., Norhaizan M.E., Liew W.P. (2018). Nutrients and Oxidative Stress: Friend or Foe?. Oxid. Med. Cell Longev..

[45] Giannakou M., Saltiki K., Mantzou E., Loukari E., Philippou G., Terzidis K., Stavrianos C., Kyprianou M., Psaltopoulou T., Karatzi K. (2018). The effect of obesity and dietary habits on oxidative stress in Hashimoto’s thyroiditis. Endocr. Connect..

[46] Sharifi-Rad M., Anil Kumar N.V., Zucca P., Varoni E.M., Dini L., Panzarini E., Rajkovic J., Tsouh Fokou P.V., Azzini E., Peluso I. (2020). Lifestyle, Oxidative Stress, and Antioxidants: Back and Forth in the Pathophysiology of Chronic Diseases. Front. Physiol..

[47] Ashok A., Andrabi S.S., Mansoor S., Kuang Y., Kwon B.K., Labhasetwar V. (2022). Antioxidant Therapy in Oxidative Stress-Induced Neurodegenerative Diseases: Role of Nanoparticle-Based Drug Delivery Systems in Clinical Translation. Antioxidants.

[48] Clemente-Suárez V.J., Martín-Rodríguez A., Redondo-Flórez L., López-Mora C., Yáñez-Sepúlveda R., Tornero-Aguilera J.F. (2023). New Insights and Potential Therapeutic Interventions in Metabolic Diseases. Int. J. Mol. Sci..

[49] Clemente-Suárez V.J., Martín-Rodríguez A., Redondo-Flórez L., Ruisoto P., Navarro-Jiménez E., Ramos-Campo D.J., Tornero-Aguilera J.F. (2023). Metabolic Health, Mitochondrial Fitness, Physical Activity, and Cancer. Cancers.

[50] Ioniță-Mîndrican C.-B., Mititelu M., Musuc A.M., Oprea E., Ziani K., Neacșu S.M., Grigore N.D., Negrei C., Dumitrescu D.-E., Mireșan H. (2022). Honey and Other Beekeeping Products Intake among the Romanian Population and Their Therapeutic Use. Appl. Sci..

[51] Mititelu M., Neacsu S.M., Oprea E., Dumitrescu D.-E., Nedelescu M., Drăgănescu D., Nicolescu T.O., Rosca A.C., Ghica M. (2022). Black Sea Mussels Qualitative and Quantitative Chemical Analysis: Nutritional Benefits and Possible Risks through Consumption. Nutrients.

[52] Catauro M., Papale F., Bollino F., Piccolella S., Marciano S., Nocera P., Pacifico S. (2015). Silica/quercetin sol–gel hybrids as antioxidant dental implant materials. Sci. Technol. Adv. Mater..

[53] Balan V., Petrache I.A., Popa M.I., Butnaru M., Barbu E., Tsibouklis J., Verestiuc L. (2012). Biotinylated chitosan-based SPIONs with potential in blood-contacting applications. J. Nanopart Res..

[54] Choi J.S., Park J.W., Park J.S. (2019). Design of Coenzyme Q10 solid dispersion for improved solubilization and stability. Int. J. Pharm..

[55] Lin Y.C., Hu S.C., Huang P.H., Lin T.C., Yen F.L. (2020). Electrospun Resveratrol-Loaded Polyvinylpyrrolidone/Cyclodextrin Nanofibers and Their Biomedical Applications. Pharmaceutics.

[56] Qin L., He Y., Zhao X., Zhang T., Qin Y., Du A. (2020). Preparation, characterization, and in vitro sustained release profile of resveratrol-loaded silica aerogel. Molecules.

[57] Yang J., Chen X., Lin J., Shen M., Wang Y., Sarkar A., Wen H., Xie J. (2024). Co-delivery of resveratrol and curcumin based on Mesona chinensis polysaccharides/zein nanoparticle for targeted alleviation of ulcerative colitis. Food Biosci..

[58] Chanphai P., Tajmir-Riahi H.A. (2017). Probing the binding of resveratrol, genistein and curcumin with chitosan nanoparticles. J. Mol. Liq..

[59] Ozon E.A., Iuga I.D.M., Mititelu M., Musuc A.M., Manolescu B.N., Petrescu S., Cusu J.P., Rusu A., Surdu V.-A., Oprea E. (2023). Pharmacotechnical, Physicochemical, and Antioxidant Evaluation of Newly Developed Capsule Formulations. Int. J. Mol. Sci..

[60] Wang Z., Hu F., Che Z.H., Song Q., Shen B.D., Yuan H.L. (2022). Preparation and in vitro release of quercetin nanocrystals self-stabilized Pickering emulsion. China J. Chin. Mater. Med..

[61] Han Q., Huang L., Luo Q., Wang Y., Wu M., Sun S., Zhang H., Wang Y. (2021). Synthesis and biological evaluation of biotin-conjugated *Portulaca oleracea* polysaccharides. RSC Adv..

[62] Zhang Z., Ge M., Wu D., Li W., Chen W., Liu P., Zhang H., Yang Y. (2024). Resveratrol-loaded sulfated Hericium erinaceus β-glucan-chitosan nanoparticles: Preparation, characterization and synergistic anti-inflammatory effects. Carbohydr. Polym..

[63] da Silva R.d.C., Teixeira J.A., Nunes W.D.G., Zangaro G.A.C., Pivatto M., Caires F.J., Ionashiro M. (2017). Resveratrol: A thermoanalytical study. Food Chem..

[64] da Costa E.M., Barbosa Filho J.M., Gomes do Nascimento T., Oliveira Macêdo R. (2002). Thermal characterization of the quercetin and rutin flavonoids. Thermochim. Acta.

[65] Lv R., Qi L., Zou Y., Zou J., Luo Z., Shao P. (2019). Preparation and structural properties of amylose complexes with quercetin and their preliminary evaluation in delivery application. Int. J. Food Prop..

[66] Council of Europe (2019). European Pharmacopoeia.

[67] Crouter A., Briens L. (2014). The effect of moisture on the flowability of pharmaceutical excipients. AAPS PharmSciTech.

[68] Sun C.C. (2007). Mechanism of moisture induced variations in true density and compaction properties of microcrystalline cellulose. Int. J. Pharm..

[69] Khan F., Pilpel N., Ingram S. (1988). The effect of moisture on the density, compaction and tensile strength of microcrystalline cellulose. Powder Technol..

[70] Koumbogle K., Gosselin R., Gitzhofer F., Abatzoglou N. (2023). Moisture Behavior of Pharmaceutical Powder during the Tableting Process. Pharmaceutics.

[71] Chaerunisaa A.Y., Sriwidodo S., Abdassah M., Ahmad U., Akhtar J. (2019). Microcrystalline Cellulose as Pharmaceutical Excipient. Pharmaceutical Formulation Design—Recent Practices.

[72] Janssen P.H.M., Depaifve S., Neveu A., Francqui F., Dickhoff B.H.J. (2021). Impact of Powder Properties on the Rheological Behavior of Excipients. Pharmaceutics.

[73] Wu Z., Wu Y., Zakhvatayeva A., Wang X., Liu Z., Yang M., Zheng Q., Wu C.-Y. (2022). Influence of moisture content on die filling of pharmaceutical powders. J. Drug Deliv. Sci. Technol..

[74] Parezanović G., Lalic-Popovic M., Golocorbin-Kon S., Todorović N., Pavlović N., Jovicic Bata J. (2019). The effect of magnesium stearate and sodium starch glycolate on powder flowability. Acta Period. Technol..

[75] Dominik M., Vraníková B., Svačinová P., Elbl J., Pavloková S., Prudilová B.B., Šklubalová Z., Franc A. (2021). Comparison of Flow and Compression Properties of Four Lactose-Based Co-Processed Excipients: Cellactose^®^ 80, CombiLac^®^, MicroceLac^®^ 100, and StarLac^®^. Pharmaceutics.

[76] Novac M., Musuc A.M., Ozon E.A., Sarbu I., Mitu M.A., Rusu A., Petrescu S., Atkinson I., Gheorghe D., Lupuliasa D. (2022). Design and Evaluation of Orally Dispersible Tablets Containing Amlodipine Inclusion Complexes in Hydroxypropyl-β-cyclodextrin and Methyl-β-cyclodextrin. Materials.

[77] Rowe R.C., Sheskey P.J., Willer P.J. (2012). Handbook of Pharmaceutical Excipients; RPS: London, UK, 2009; pp. 651–653, 2. Limin Shi, Sayantan Chattoraj, Changquan Calvin Sun, Reproducibility of flow properties of microcrystalline cellulose—Avicel PH102. Powder Technol..

[78] Pop A.L., Crișan S., Bârcă M., Ciobanu A.-M., Varlas V.N., Pop C., Pali M.-A., Cauni D., Ozon E.A., Udeanu D. (2021). Evaluation of Dissolution Profiles of a Newly Developed Solid Oral Immediate-Release Formula Containing Alpha-Lipoic Acid. Processes.

[79] Pop A.L., Henteș P., Pali M.A., Oșanu L., Ciobanu A.M., Nasui B.A., Mititelu M., Crișan S., Peneș O.N. (2022). Study regarding a new extended-release calcium ascorbate and hesperidin solid oral formulation. Farmacia.

[80] Stranzinger S., Faulhammer E., Calzolari V., Biserni S., Dreu R., Šibanc R., Paudel A., Khinast J.G. (2017). The effect of material attributes and process parameters on the powder bed uniformity during a low-dose dosator capsule filling process. Int. J. Pharm..

[81] Liu Y., Liu C., Li J. (2020). Comparison of Vitamin C and Its Derivative Antioxidant Activity: Evaluated by Using Density Functional Theory. ACS Omega.

[82] Carrillo-Martinez E.J., Flores-Hernández F.Y., Salazar-Montes A.M., Nario-Chaidez H.F., Hernández-Ortega L.D. (2024). Quercetin, a Flavonoid with Great Pharmacological Capacity. Molecules.

[83] García-García F.J., Monistrol-Mula A., Cardellach F., Garrabou G. (2020). Nutrition, Bioenergetics, and Metabolic Syndrome. Nutrients.

[84] Pallotti F., Bergamini C., Lamperti C., Fato R. (2021). The Roles of Coenzyme Q in Disease: Direct and Indirect Involvement in Cellular Functions. Int. J. Mol. Sci..

[85] Rizzardi N., Liparulo I., Antonelli G., Orsini F., Riva A., Bergamini C., Fato R. (2021). Coenzyme Q10 Phytosome Formulation Improves CoQ10 Bioavailability and Mitochondrial Functionality in Cultured Cells. Antioxidants.

[86] Farhan M., Rizvi A. (2023). The Pharmacological Properties of Red Grape Polyphenol Resveratrol: Clinical Trials and Obstacles in Drug Development. Nutrients.

[87] Pezzuto J.M. (2008). Resveratrol as an Inhibitor of Carcinogenesis. Pharm. Biol..

[88] Aghababaei F., Hadidi M. (2023). Recent Advances in Potential Health Benefits of Quercetin. Pharmaceuticals.

[89] Nam J.-S., Sharma A.R., Nguyen L.T., Chakraborty C., Sharma G., Lee S.-S. (2016). Application of Bioactive Quercetin in Oncotherapy: From Nutrition to Nanomedicine. Molecules.

[90] Kim J.H., Park E.Y., Ha H.K., Jo C.M., Lee W.J., Lee S.S., Kim J.W. (2016). Resveratrol-loaded Nanoparticles Induce Antioxidant Activity against Oxidative Stress. Asian-Australas. J. Anim. Sci..

[91] Giordano M.E., Lionetto M.G. (2023). Intracellular Redox Behavior of Quercetin and Resveratrol Singly and in Mixtures. Molecules.

[92] Skroza D., Mekinić I.G., Svilović S., Šimat V., Katalinić V. (2015). Investigation of the potential synergistic effect of resveratrol with other phenolic compounds: A case of binary phenolic mixtures. J. Food Compos. Anal..

[93] Joshi T., Deepa P.R., Sharma P.K. (2022). Effect of Different Proportions of Phenolics on Antioxidant Potential: Pointers for Bioactive Synergy/Antagonism in Foods and Nutraceuticals. Proc. Natl. Acad. Sci. India Sect. B Biol. Sci..

[94] Gherardi G., Corbioli G., Ruzza F., Rizzuto R. (2022). CoQ_10_ and Resveratrol Effects to Ameliorate Aged-Related Mitochondrial Dysfunctions. Nutrients.

[95] Yu T., Wang L., Zhang L., Deuster P.A. (2023). Mitochondrial Fission as a Therapeutic Target for Metabolic Diseases: Insights into Antioxidant Strategies. Antioxidants.

[96] Hsu M.Y., Hsiao Y.P., Lin Y.T., Chen C., Lee C.M., Liao W.C., Tsou S.C., Lin H.W., Chang Y.Y. (2021). Quercetin Alleviates the Accumulation of Superoxide in Sodium Iodate-Induced Retinal Autophagy by Regulating Mitochondrial Reactive Oxygen Species Homeostasis through Enhanced Deacetyl-SOD2 via the Nrf2-PGC-1α-Sirt1 Pathway. Antioxidants.

[97] Agrawal S., Agrawal A., Said H.M. (2016). Biotin deficiency enhances the inflammatory response of human dendritic cells. Am. J. Physiol. Cell Physiol..

[98] Cirilli I., Damiani E., Dludla P.V., Hargreaves I., Marcheggiani F., Millichap L.E., Orlando P., Silvestri S., Tiano L. (2021). Role of Coenzyme Q_10_ in Health and Disease: An Update on the Last 10 Years (2010–2020). Antioxidants.

[99] Meng T., Xiao D., Muhammed A., Deng J., Chen L., He J. (2021). Anti-Inflammatory Action and Mechanisms of Resveratrol. Molecules.

[100] Chang K.-H., Chen C.-M. (2020). The Role of Oxidative Stress in Parkinson’s Disease. Antioxidants.

[101] da Rosa M.M., de Amorim L.C., de Oliveira Alves J.V., da Silva Aguiar I.F., da Silva Oliveira F.G., da Silva M.V., dos Santos M.T.C. (2022). The promising role of natural products in Alzheimer’s disease. Brain Disord..

[102] Pritam P., Deka R., Bhardwaj A., Srivastava R., Kumar D., Jha A.K., Jha N.K., Villa C., Jha S.K. (2022). Antioxidants in Alzheimer’s Disease: Current Therapeutic Significance and Future Prospects. Biology.

[103] Cronin K., Ring D., Sheehan L., Foulon A. (2015). Probabilistic analysis of weight variability in tablets & capsules arising from the filling of a cavity with powder of a poly-dispersed size. Powder Technol..

[104] Rowe R.C., York P., Colbourn E.A., Roskilly S.J. (2005). The influence of pellet shape, size and distribution on capsule filling—A preliminary evaluation of three-dimensional computer simulation using a Monte-Carlo technique. Inter. J. Pharm..

[105] Coc L.M.C., Lacatusu I., Badea N., Penes O., Cobelschi C.P., Pop A., Meghea A. (2022). Curcumin co-loaded with a lipid mediator in the same nanostructured lipid delivery system. Farmacia.

[106] Pop A.L., Musuc A.M., Nicoară A.C., Ozon E.A., Crisan S., Penes O.N., Nasui B.A., Lupuliasa D., Secăreanu A.A. (2022). Optimization of the Preformulation and Formulation Parameters in the Development of New Extended-Release Tablets Containing Felodipine. Appl. Sci..

[107] Balaci T., Velescu B., Karampelas O., Musuc A.M., Nitulescu G.M., Ozon E.A., Nitulescu G., Gird C.E., Fita C., Lupuliasa D. (2021). Physico-Chemical and Pharmaco-Technical Characterization of Inclusion Complexes Formed by Rutoside with beta-Cyclodextrin and Hydroxypropyl-beta-Cyclodextrin Used to Develop Solid Dosage Forms. Processes.

[108] Mitu M.A., Cretu E.A., Novac M., Karampelas O., Nicoara A., Nitulescu G., Lupuleasa D., Draganescu D., Arsene A. (2017). The Flowing Characteristics of Some Composed Powders Containing Inclusion Complexes in Beta-Cyclodextrin. 17th Romanian National Congress of Pharmacy: 21st Century Pharmacy—Between Intelligent Specialization and Social Responsibility 2018, Bucharest, Romania, 26–29 September 2018.

[109] Hoag S.W., Qiu Y., Chen Y., Zhang G., Yu L., Mantri R. (2017). Chapter 27—Capsules Dosage Form: Formulation and Manufacturing Considerations. Developing Solid Oral Dosage Forms.

[110] Re R., Pellegrini N., Proteggente A., Pannala A., Yang M., Rice-Evans C. (1999). Antioxidant activity applying an improved ABTS radical cationic decolorization assay. Free Radic. Biol. Med..

[111] Margina D., Olaru O.T., Ilie M., Grădinaru D., Guțu C., Voicu S., Dinischiotu A., Spandidos D.A., Tsatsakis A.M. (2015). Assessment of the potential health benefits of certain total extracts from Vitisvinifera, Aesculushyppocastanum and Curcuma longa. Exp. Ther. Med..

[112] Nair V.D.P., Dairam A., Agbonon A., Arnason J.T., Foster B.C., Kanfer I. (2007). Investigation of the antioxidant activity of African potato (*Hypoxis hemerocallidea*). J. Agric. Food Chem..

[113] Popovici V., Matei E., Cozaru G.C., Bucur L., Gîrd C.E., Schröder V., Ozon E.A., Mitu M.A., Musuc A.M., Petrescu S. (2022). Design, Characterization, and Anticancer and Antimicrobial Activities of Mucoadhesive Oral Patches Loaded with *Usnea barbata* (L.) F. H. Wigg Ethanol Extract F-UBE-HPMC. Antioxidants.

